# Nonreciprocal charge transport at topological insulator/superconductor interface

**DOI:** 10.1038/s41467-019-10658-3

**Published:** 2019-06-21

**Authors:** Kenji Yasuda, Hironori Yasuda, Tian Liang, Ryutaro Yoshimi, Atsushi Tsukazaki, Kei S. Takahashi, Naoto Nagaosa, Masashi Kawasaki, Yoshinori Tokura

**Affiliations:** 1 Department of Applied Physics and Quantum-Phase Electronics Center (QPEC), University of Tokyo, Tokyo, 113-8656 Japan; 2 RIKEN Center for Emergent Matter Science (CEMS), Wako, 351-0198 Japan; 3 Institute for Materials Research, Tohoku University, Sendai, 980-8577 Japan; 4 Tokyo College, University of Tokyo, Tokyo, 113-8656 Japan; 5 Present Address: Department of Physics, Massachusetts Institute of Technology, Cambridge, MA 02139 USA

**Keywords:** Superconducting properties and materials, Surfaces, interfaces and thin films, Topological insulators, Superconducting devices

## Abstract

Topological superconductor is attracting growing interest for its potential application to topological quantum computation. The superconducting proximity effect on the topological insulator surface state is one promising way to yield topological superconductivity. The superconductivity realized at the interface between Bi_2_Te_3_ and non-superconductor FeTe is one such candidate. Here, to detect the mutual interaction between superconductivity and topological surface state, we investigate nonreciprocal transport; i.e., current-direction dependent resistance, which is sensitive to the broken inversion symmetry of the electronic state. The largely enhanced nonreciprocal phenomenon is detected in the Bi_2_Te_3_/FeTe heterostructure associated with the superconducting transition. The emergent nonreciprocal signal at low magnetic fields is attributed to the current-induced modulation of supercurrent density under the in-plane magnetic fields due to the spin-momentum locking. The angular dependence of the signal reveals the symmetry of superconductivity and indicates the existence of another mechanism of nonreciprocal transport at high fields.

## Abstract (web summary)

The superconducting proximity effect on the surface state of a topological insulator is promising to generate topological superconductivity. Here, Yasuda et al. reported enhanced nonreciprocal charge transport in a Bi_2_Te_3_/FeTe heterostructure with an emerging superconducting order parameter.

## Introduction

Topological superconductivity (TSC) is extensively sought for in contemporary condensed matter physics^1–3^. In a conventional superconductor, spin-up and spin-down electrons form a spin-singlet Cooper pair, and even-parity *s*-wave superconductor is realized. However, in a spinless superconductor, i.e., paired systems with only one active fermionic species, an odd-parity *p* + *ip*-wave superconductor is induced because of Pauli’s exclusion principle. Such a superconductor is topologically non-trivial and supports Majorana zero modes at the boundary or at the vortex core, which can act as building blocks of topological quantum computation owing to their non-abelian statistics^1–3^. To realize a non-degenerate spinless mode, one has to fight against the Kramers degeneracy: One way to deal with the Kramars degeneracy is time-reversal symmetry breaking as discussed in superfluid ^3^He-A phase^4^ and superconducting Sr_2_RuO_4_^5^. Another strategy is to utilize spin-splitting by inversion symmetry breaking as proposed in the synthetic topological superconductors, such as superconducting–proximity coupled Rashba wires^6–8^ and topological insulator (TI) surface states^9–14^. Spin splitting caused by relativistic spin–orbit coupling and the inversion symmetry breaking yields a spin non-degenerate band, as exemplified by the spin–momentum-locked topological surface state. In these systems, the understanding of the superconducting property on the spin-splitted band structure is a prerequisite for the realization of TSC.

Recently, nonreciprocal charge transport measurement has been developed as a new probe to detect the spin-splitting in inversion symmetry-broken systems^15–24^. Reflecting the asymmetric nature of the electronic band structure with spin splitting, the electrical resistivity is expected to vary depending on the current and magnetic field direction. For example, nonreciprocal resistance is experimentally observed in materials with Rashba-type spin splitting, such as a bulk Rashba semiconductor^15^, interfaces^16–19^, and TI surfaces^20–22^. Under the in-plane magnetic field perpendicular to the current, the resistance value depends on the spin accumulation direction, either parallel or antiparallel to the external magnetic field^15,18–22^. Hence, this method works as an alternative to angle-resolved photoemission spectroscopy (ARPES)^13^ and scanning tunneling microscope (STM)^14^ to discuss the effect of spin splitting via transport measurement. Furthermore, nonreciprocal transport is also observed in non-centrosymmetric superconductors, such as a chiral nanotube^23^ and a transition metal dichalcogenide^24^, demonstrating that this probe is also applicable to inversion symmetry broken superconductors.

**Fig. 1:**
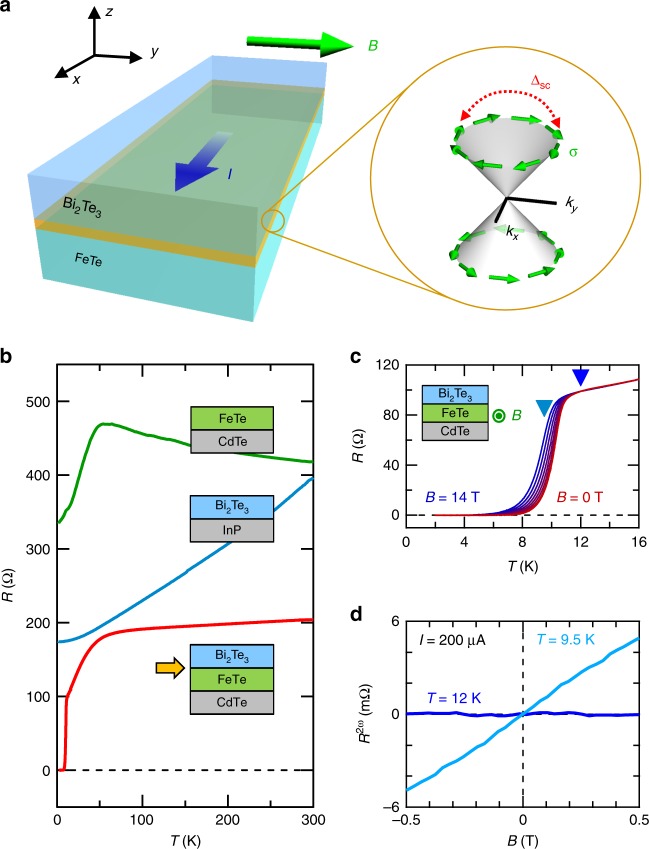
Nonreciprocal transport in a Bi_2_Te_3_/FeTe heterostructure. **a** The illustration of a Bi_2_Te_3_/FeTe heterostructure (Bi_2_Te_3_: blue, FeTe: green). The interface of Bi_2_Te_3_ and FeTe shows superconductivity (yellow region). The nonreciprocal transport is measured with current *I* along *x*-direction under the in-plane magnetic field *B* (along *y*-direction) perpendicular to the current. The mutual interaction between spin–momentum-locked topological surface state and superconductivity (as represented by *Δ*_SC_) is expected at the interface as expressed in momentum space in the right circle. *σ* represents the spin direction of the electron. **b** Temperature dependence of resistance in FeTe(18 nm)/CdTe(100) (green), Bi_2_Te_3_(15 nm)/InP(111) (blue) and Bi_2_Te_3_(15 nm)/FeTe(18 nm)/CdTe(100) (red) thin films. The superconductivity and topological surface state appear at the interface between Bi_2_Te_3_ and FeTe as denoted by the yellow allow. **c** The temperature dependence of resistance in Bi_2_Te_3_/FeTe heterostructure under in-plane magnetic field *B* = 0, 2, 4, 6, 8, 10, 12, and 14 T. The resistance is measured under *I* = 1 μA. **d** The magnetic field dependence of *R*^2*ω*^ in Bi_2_Te_3_/FeTe heterostructure at *T* = 12 K (blue, normal state) and at *T* = 9.5 K (light blue, below the superconducting onset temperature) measured with a current magnitude of *I* = 200 μA

In this study, we target the emergent superconductivity at the interface between Bi_2_Te_3_ and FeTe^25^. The interface superconductivity is induced when a three-dimensional TI Bi_2_Te_3_^26^ is grown on a parent compound of iron-based superconductor FeTe as schematically shown in Fig. 1a. Since the topological surface state locates at the interface, the interaction between them makes this system a strong candidate of TSC. However, it has remained unclear how the spin–momentum locking of the surface state affects the superconducting property^25^. To study the interplay between them, we here employ nonreciprocal charge transport. The nonreciprocal transport is especially effective to this system because surface-sensitive methods, such as ARPES and STM is not applicable to such a buried interface. The large nonreciprocal transport is observed to be associated with the superconducting transition of Bi_2_Te_3_/FeTe. This is revealed to originate from the current-induced-modulation of supercurrent density due to spin–momentum locking, which represents the close connection between superconductivity and topological surface state.

## Result

### Sample and basic characterization

We grew the heterostructure of Bi_2_Te_3_/FeTe using molecular-beam epitaxy (MBE) on CdTe(100) substrate. Van der Waals nature of Bi_2_Te_3_ and FeTe allows the sharp interface despite the difference in the lattice symmetry. The scanning transmission electron microscopy and energy-dispersive x-ray spectroscopy images demonstrate the high quality of the interface without discernable interdiffusion (see Supplementary Fig. 1 and Supplementary Note 1). Figure 1b shows the temperature dependence of resistance for the respective samples, FeTe(18 nm), Bi_2_Te_3_(15 nm), and Bi_2_Te_3_(15 nm)/FeTe(18 nm) bilayer thin films. FeTe thin film shows a semi-metallic behavior with a kink at around 50 K associated with the transition to the bicollinear antiferromagnetic state^27^, while Bi_2_Te_3_ shows a metallic behavior due to its electron-doped nature. The resistance of Bi_2_Te_3_/FeTe behaves as parallel conduction of these two materials except for the resistance drop to zero at around *T*_c0_ = 10.7 K (Fig. 1c), indicating the appearance of superconductivity at the interface^25^. The fitting of the temperature dependence of resistivity with the Berezinskii–Kosterlitz–Thouless (BKT) transition^28–30^ and the jump in the power law of current–voltage characteristics (Supplementary Fig. 2 and Supplementary Note 2) verifies the two-dimensional nature of superconductivity^25^, where the binding of the vortex–antivortex pairs realizes the zero-resistance state. Because of the two-dimensional nature of superconductivity, the superconducting order parameter appears at *T*_c0_, but finite resistance exists in the intermediate state because of the current-induced motion of vortex and antivortex. When vortex and antivortex form a pair at *T*_BKT_, zero-resistance state, i.e., the superconducting state is realized.

### Nonreciprocal transport in Bi_2_Te_3_/FeTe

If inversion symmetry of the electronic system is broken, the electrical voltage *V* under the in-plane magnetic field is phenomenologically described up to a linear order in the magnetic field *B* and second order of current *I* as^16^

1 $$V = R_0I(1 + \gamma \,({\bf{B}} \times {\bf{z}}) \cdot {\bf{I}}).$$

Here, we define the direction normal to the plane as *z*-axis and *γ* is a coefficient representing the nonlinear and nonreciprocal transport. When the current is perpendicular to the in-plane magnetic field, Eq. (1) becomes *V* = *R*_0_*I* + *γR*_0_*BI*^2^. If the second term is finite, the resistance value depends on the current direction, namely the nonreciprocal transport appears. Since the second term is proportional to *I*^2^, measurements of the second harmonic resistance $R^{2\omega } = \frac{{R_0}}{{\sqrt 2 }}\gamma BI$ under ac current can detect the nonreciprocal transport (see Supplementary Note 3 for the derivation). We fabricated a Hall bar of 100 μm width and measured *R*^2*ω*^ with a lock-in amplifier^18–24^ under the ac current of *I* = 200 μA (*i* = 2 A/m in current density) unless otherwise noted. Figure 1d displays the magnetic field dependence of *R*^2*ω*^ in the normal and the intermediate states. In the normal state (*T* = 12 K), *R*^2*ω*^ is almost zero. On the other hand, *B*-linear *R*^2*ω*^ appears at *T* = 9.5 K in the intermediate region as the superconducting order parameter develops.

**Fig. 2:**
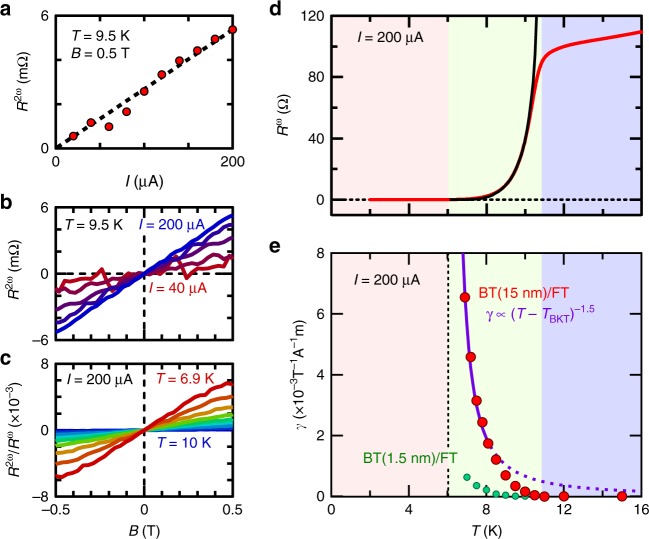
Current-magnitude and temperature dependence of nonreciprocal transport. **a** The current-magnitude dependence of second harmonic resistance *R*^2ω^ at *T* = 9.5 K, and *B* = 0.5 K deduced from the data shown in **b**. The black dotted line is the fitting line. **b** The magnetic field dependence of *R*^2ω^ at *T* = 9.5 K measured under *I* = 40, 80, 120, 160, and 200 μA. **c** The magnetic field dependence of *R*^2ω^/*R*^ω^ measured under *I* = 200 μA at *T* = 6.9, 7.2, 7.5, 7.8, 8.1, 8.5, 9, 9.5, and 10 K. **d** The temperature dependence of resistance measured under *I* = 200 μA. The black curve is the fitting of the Berezinskii–Kosterlitz–Thouless (BKT) transition using Halperin–Nelson formula, $R = R_0\,{\mathrm{exp}}\left( { - 2b\left( {\frac{{T_{{\mathrm{c}}0} - T}}{{T - T_{{\mathrm{BKT}}}}}} \right)^{0.5}} \right)$, where *R*_0_ and *b* are material parameters. *T*_c0_ is the temperature at which the finite amplitude of the order parameter develops and *T*_BKT_ is the BKT transition temperature. The fitting gives the values, *T*_c0_ = 10.7 K and *T*_BKT_ = 6.0 K. The blue, green, and red regions correspond to normal, intermediate, and superconducting regions, respectively. **e** The temperature dependence of *γ*-value measured under *I* = 200 μA derived from **c**. The red points are the measurement on Bi_2_Te_3_(15 nm)/FeTe(18 nm) sample (denoted as BT(15 nm)/FT). The green point are the measurement on Bi_2_Te_3_(1.5 nm)/FeTe(18 nm) sample (denoted as BT(1.5 nm)/FT). Note that all the measurements, except for Fig. 1b, and the green curve of Fig. 2e, are done on the BT(15 nm)/FT sample. The purple curve is the fitting of the red points with the formula *γ* = *β*$(T - T_{{\mathrm{BKT}}})^{ - 1.5}$, where *β* = 5.3 × 10^−3^ T^−1^ A^−1^ m. Note that the BKT model and the fitting is valid only at around *T*_BKT_, which is represented by the solid purple curve. The purple dotted curve is out of the applicable range of theory

In contrast to the first harmonic resistance *R*^*ω*^, which is independent of the current magnitude, *R*^2*ω*^ is proportional to the current as shown in Fig. 2a, b, which is consistent with Eq. (1). Thus, the *γ* value can be derived from the slope of the magnetic field dependence of *R*^2*ω*^/*R*^*ω*^ as shown in Fig. 2c. To see the correspondence between the temperature dependence of *R*^*ω*^ and *γ* value (Fig. 2d, e), we categorize them into three regions: normal, intermediate, and superconducting region. In the normal region, *R*^2*ω*^ is not observed within the measurement noise level. In the superconducting region, *R*^2*ω*^ vanishes with the disappearance of *R*^*ω*^ (see Supplementary Fig. 3 and Supplementary Note 4). On the other hand, *γ* becomes finite in the intermediate region. Especially, *γ* value shows a divergent behavior towards the BKT transition temperature, *T*_BKT_, since *R*^2*ω*^ drops to zero more slowly than *R*^*ω*^. The maximum of *γ* is 6.5 × 10^−3^ T^−1^ A^−1^ m at *T* = 6.9 K, which is more than five orders of magnitude larger than the reported value of *γ* = 6 × 10^−8^ T^−1^ A^−1^ m in TI without superconductivity^22^. The large enhancement of *γ* associated with the superconducting transition can be qualitatively explained as follows: The energy scale governing the system changes from the Fermi energy *E*_F_ (~a few hundred meV) to the much smaller superconducting gap Δ_SC_ (~1 meV) along with the superconducting transition. Consequently, the relative energy scale of spin–orbit interaction and magnetic field gets larger, resulting in the enhancement of nonreciprocal transport^24^.

### Surface state origin of nonreciprocal transport

Because of the following two reasons, it is natural to guess that topological surface state plays a vital role in nonreciprocal transport: (i) spin–orbit coupling is an essential source of nonreciprocal transport^31^ and (ii) topological surface state with strong spin–orbit coupling coexists with superconductivity at the interface where nonreciprocal transport appears. To substantiate this argument, we study the nonreciprocal transport of Bi_2_Te_3_(1.5 nm)/FeTe(18 nm) with much thinner Bi_2_Te_3_ thickness. Although the normal state resistance and the BKT transition temperature is almost the same in the two samples (Supplementary Figs 4 and 5, and Supplementary Notes 5 and 6), the *γ*-value for Bi_2_Te_3_(1.5 nm)/FeTe(18 nm) is about an order of magnitude smaller than that of Bi_2_Te_3_(15 nm)/FeTe(18 nm) as shown in the green points in Fig. 2e. This dramatic change is attributed to the suppressed spin polarization of the surface state in the ultrathin limit because of the hybridization between the top and surface states^32,33^. Thus, the large suppression of the signal points to the surface state origin of nonreciprocal transport.

On the basis of the above observation, we theoretically treat the superconducting-proximity-coupled surface state to discuss the temperature dependence of *γ*. A recent theoretical study^34^ suggests that spin–momentum locking of the surface state causes the renormalization of supercurrent density under the current in superconducting-proximity-coupled TI. As a result, the BKT transition temperature is modulated as *T*′_BKT_ = *T*_BKT_(1 + *αBI*). Interestingly, this equation means that resistance in this system can decrease for a specific current direction, different from the conventional superconductors, where resistance increases by the excitation with large current. By substituting *T*′_BKT_ to the standard BKT model which is valid at around BKT transition temperature, we get

2 $$R 	= R_1{\mathrm{exp}}( - b(T - T\prime _{{\mathrm{BKT}}})^{ - 0.5})\\ 	= R_1{\mathrm{exp}}\left( \!{ - b(T - T_{{\mathrm{BKT}}})^{ - 0.5}} \right)\left( {1 - 0.5\alpha bT_{{\mathrm{BKT}}}(T - T_{{\mathrm{BKT}}}\!)^{ - 1.5}BI} \right).$$

Here, we employed perturbative expansion about *αBI*, which is justified because *R*^2*ω*^ is several orders of magnitude smaller than *R*^*ω*^ as shown in Fig. 2c. Comparing the result with Eq. (1), the *γ*-value is expected to diverge as (*T* − *T*_BKT_)^−1.5^ ^34^. In fact, the fitting of the *γ-*value by the formula (*T* − *T*_BKT_)^−1.5^ in Fig. 2e well reproduces the divergent behavior toward *T*_BKT_ (see Supplementary Fig. 7 and Supplementary Note 8 for the reproducibility in another sample). The good consistency between the theory and the experiment supports that the enhancement of nonreciprocal transport can be understood in terms of the current-induced modulation of supercurrent density in the superconducting-proximity-coupled surface state.

### High field behavior and angular dependence of nonreciprocal transport

**Fig. 3:**
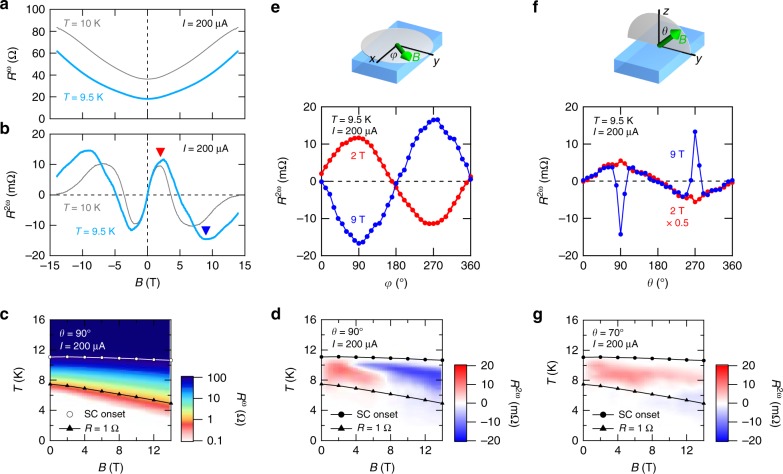
Nonreciprocal transport at high magnetic fields. **a** The magnetic field dependence of first harmonic resistance *R*^ω^ at *T* = 9.5 K (light blue) and *T* = 10 K (gray) measured under *I* = 200 μA. **b** The magnetic field dependence of second harmonic resistance *R*^2*ω*^. at *T* = 9.5K and *T* = 10 K measured under *I* = 200 μA. The red and blue triangles represent the positive and negative peaks at around *B* = 2 T and *B* = 9 T, respectively. **c** The contour plot of *R*^*ω*^ in the plane of magnetic field and temperature at *θ* = 90° (*B* || *y*, defined in **f**) measured under *I* = 200 μA. The superconducting onset and *R* = 1 Ω are shown in circle and triangle, respectively. **d** The contour plot of *R*^2*ω*^ in the plane of magnetic field and temperature at *θ* = 90° measured under *I* = 200 μA. **e** The in-plane magnetic-field direction dependence of *R*^2*ω*^ at *B* = 2 T (red) and *B* = 9 T (blue) within *xy* plane measured under *I* = 200 μA. *φ* is defined as an angle in *xy* plane measured from the *x-*axis. **f** The out-of-plane magnetic-field direction dependence of *R*^2*ω*^ at *B* = 2 T (red) and *B* = 9 T (blue) within *zy* plane measured under *I* = 200 μA. *θ* is defined as an angle in *zy* plane measured from the *z-*axis. **g** The contour plot of *R*^2*ω*^ in the plane of magnetic field and temperature at *θ* = 70° measured under *I* = 200 μA

In the above, we have considered the second harmonic resistance at a low magnetic field region, we next discuss *R*^2*ω*^ at higher fields. Figure 3a, b show the magnetic field dependence of *R*^*ω*^ and *R*^2*ω*^ for *T* = 9.5 and 10 K. The breaking of superconductivity with the application of the magnetic field is clearly discerned in Fig. 3a. At low magnetic fields, the linear-positive slope of *R*^2*ω*^ with respect to the magnetic field is observed. The positive slope can be explained in terms of the modulation of supercurrent density as discussed earlier. At high magnetic fields, however, *R*^2*ω*^ shows a sign reversal. With the further application of the magnetic field, *R*^2*ω*^ goes to zero along with the breaking of superconductivity, which is clearly discerned at *T* = 10 K at around *B* = 14 T. The *B*–*T* diagrams for *R*^*ω*^ and *R*^2*ω*^ are displayed as contour plots in Fig. 3c, d, respectively. We can again see that *R*^2*ω*^ appears positively at low fields and negatively at high fields at the intermediate temperature region. To discuss the origin of the negative component, we investigate the magnetic field direction dependence of *R*^2*ω*^. Figure 3e, f show the *xy* plane (in-plane) and *zy* plane (out-of-plane) magnetic-field directional dependence of *R*^2*ω*^, respectively. *R*^2*ω*^ becomes almost zero at *B*||*x* (*φ* = 0°, 180°, 360°) and *B*||*z* (*θ* = 0°, 180°, 360°), consistent with the symmetry of a superconducting-proximity-coupled TI^20–22^ (see Supplementary Fig. 9 and Supplementary Discussion 1 for the discussion on the disappearance of *R*^2*ω*^ under *B*||*z*). In *xy* plane (Fig. 3e), *R*^2*ω*^ shows sin *φ* dependence both at 2 and 9 T, meaning that the signal is scaled with the *y* component of the magnetic field, consistent with Eq. (1). Similarly, in the *zy* plane (Fig. 3f), *R*^2*ω*^ shows sin*θ* behavior at 2 T, and the signal at 9 T basically follows sin*θ* behavior with the same sign as the 2 T signal. Remarkably, however, a sudden sign reversal of the signal is observed at *θ* = 90° and 270°, namely the negative component appears only when *B* is almost exactly aligned to in-plane (see Supplementary Figs. 6 and 8, and Supplementary Notes 7 and 8 for further analysis and reproducibility). In fact, the negative component almost vanishes at *θ* = 70°, leaving only the positive component, as shown in Fig. 3g. These results imply that the positive and the negative components are governed by the different origins. The sudden appearance of the negative component under the in-plane magnetic field indicates that it originates from the in-plane magnetic vortex along the *y*-direction^35^. The current-induced renormalization of supercurrent density may result in the modulation of the nucleation/annihilation energy barrier of the in-plane vortex, which can appear as nonreciprocal resistance^35^. Further theoretical and experimental investigation is required to fully reveal the origins of the negative component.

## Discussion

The large nonreciprocal charge transport in the superconducting interface of Bi_2_Te_3_/FeTe can be utilized as a magnetically controllable superconducting diode^24^ and as a rectenna at low temperatures^35^. The nonreciprocal transport can be well-explained by the modulation of supercurrent density due to spin–momentum locking, exemplifying the close connection between superconductivity and topological surface state. Thus, tuning the Fermi level to the surface state^36^ will make the present system a desirable platform to study TSC and the associated formation of Majorana fermion. The nonreciprocal transport measurement is also applicable to other TSC candidates, such as LaAlO_3_/SrTiO_3_ interface^37,38^, superconducting-proximity coupled Rashba wire^7,8^, and topological surfaces^10–14^, which will help to discuss the effect of spin splitting on the superconducting properties.

During the final revision of the manuscript, we noticed the independent ARPES study of Bi_2_Te_3_/FeTe, which suggests that charge transfer from FeTe to Bi_2_Te_3_ induces the interfacial superconductivity in hole-doped FeTe^39^. The interfacial superconductivity induces the proximity effect on the surface state of Bi_2_Te_3_, which is in line with the discussion of our present study.

## Methods

### MBE thin film growth

Thin films were grown by MBE on insulating CdTe(100) and InP(111) substrates. The growth temperatures for FeTe and Bi_2_Te_3_ were 400 and 180 °C, respectively. CdTe(100) substrates were etched with bromine–methanol (bromine 0.01%) for 5 min before the deposition. For FeTe, the flux ratio was fixed at Fe:Te = 1:30 and the growth rate was about 0.7 nm/min. For Bi_2_Te_3_, the flux ratio was fixed at Bi:Te = 1:2 and the growth rate was about 0.2 nm/min. To suppress the degradation of the film, AlO_*x*_ capping layer with a thickness of about 3 nm was deposited at room temperature with an atomic layer deposition system immediately after taking out the samples from the MBE vacuum chamber.

### Device fabrication

The Hall-bar device pattern was defined by a photolithography technique and wet etching processes with HCl:H_3_PO_4_:H_2_O = 1:1:8. For Ohmic-contact electrodes, 5 nm Ti/45 nm Au were deposited with an electron-beam evaporator.

### Transport measurements

The first and second harmonic resistances, *R*^*ω*^ and *R*^2*ω*^, were measured using a current source (Keithley: Model 6221) and lock-in amplifiers (SRS: SR830). The measurement current and frequency were fixed at 200 μA (root mean square) unless otherwise noted and at 13 Hz, respectively. The measurements were done in Physical Property Measurement System (Quantum Design: PPMS). *R*^2*ω*^ was anti-symmetrized with respect to *B*.

## Supplementary information

Supplementary Information

Peer Review File

## Data Availability

The data that support plots within this paper and other findings of this study are available from the corresponding author upon reasonable request.
