# Supplementary material for: Nonreciprocal charge transport at topological insulator/superconductor interface
Source: Nat Commun. 2019 Jun 21;10:2734. doi: 10.1038/s41467-019-10658-3 (PMC6588591; doi:10.1038/s41467-019-10658-3)
Supplement: Supplementary file 1 — Supplementary Information [file 41467_2019_10658_MOESM1_ESM.pdf]

Supplementary Information for

Nonreciprocal charge transport at topological insulator/superconductor interface

Kenji Yasuda, Hironori Yasuda, Tian Liang, Ryutaro Yoshimi, Atsushi Tsukazaki,

Kei S. Takahashi, Naoto Nagaosa, Masashi Kawasaki, Yoshinori Tokura

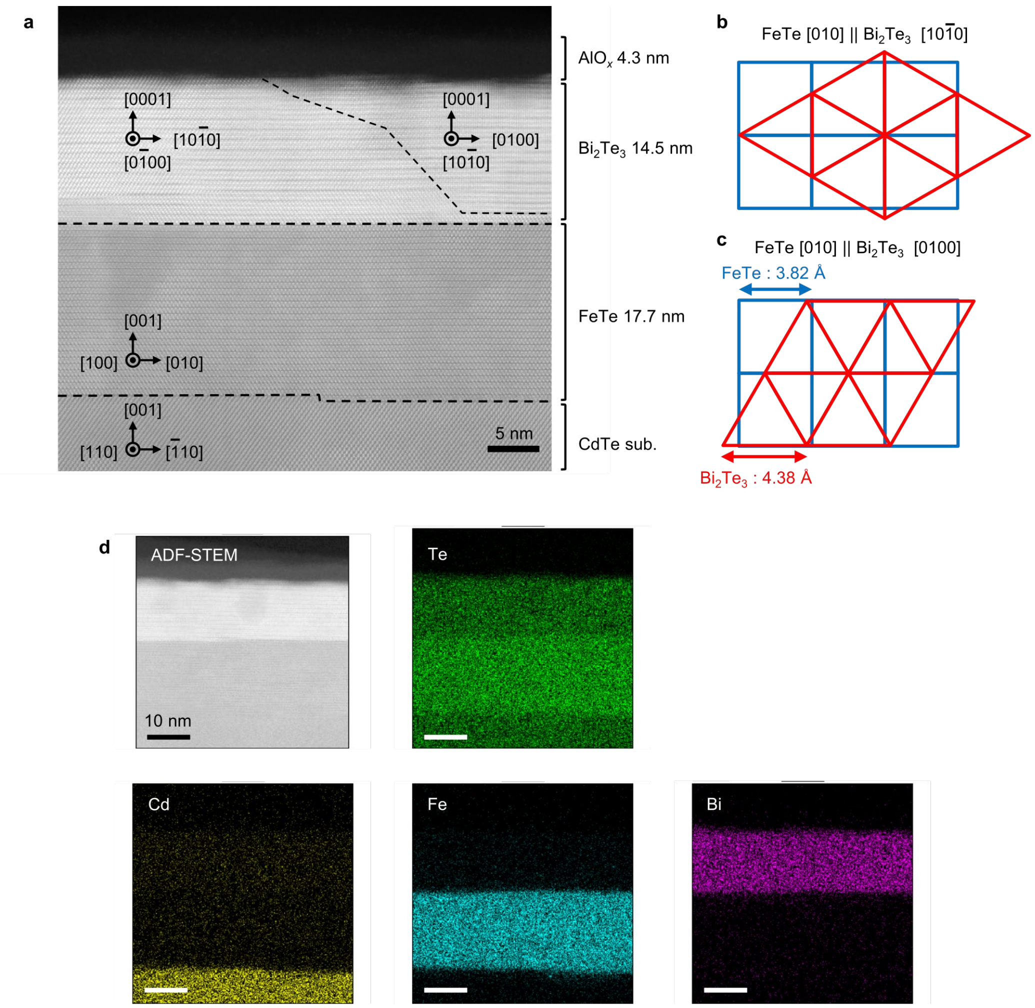

Supplementary Figure 1 | Structural characterization of Bi_2Te_3(15 nm)/FeTe(18 nm)/CdTe. **a**, Cross-sectional high-angle annular dark-field scanning transmission electron microscopy (HAADF-STEM) image of a heterostructure Bi_2Te_3(15 nm)/FeTe(18 nm)/CdTe capped with AlO_x. Two types of domains of Bi_2Te_3 with $[0\bar{1}00]$ axis along the vertical direction to the sheet and $[10\bar{1}0]$ axis along the vertical direction to the sheet is observed. **b** and **c**, The illustration of the two possible configurations of alignment of Bi_2Te_3 with three-fold symmetry on FeTe with four-fold symmetry seen from *c*-axis. **d**, Distribution maps of each elements, Te, Cd, Fe and Bi studied by an energy dispersive x-ray spectroscopy (EDX) for the area shown in HAADF-STEM image.

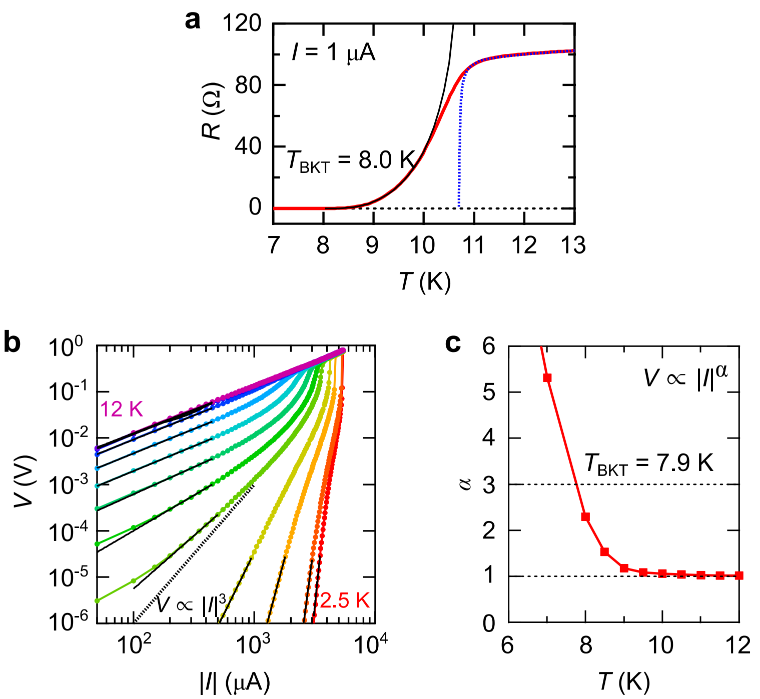

Supplementary Figure 2 | Characterization of superconductivity. a, The temperature dependence of resistance measured with the injection of I = 1 μA. The dotted blue curve is the fitting curve by the Aslamazov–Larkin equation. The solid black curve is the fitting curve by the Halperin-Nelson equation. **b,** A log-log plot of current-voltage (I-V) characteristics. The measurement temperatures are T = 2.5, 4, 6, 7, 8, 8.5, 9, 9.5, 10, 10.5, 11, 11.5 and 12 K. The dotted line corresponds to the $V \propto |I|^3$ behavior. **c,** The temperature dependence of α value obtained from the fitting (the black lines) in b.

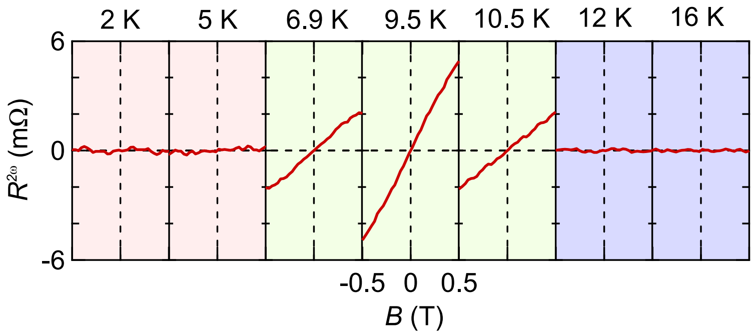

Supplementary Figure 3 | Temperature dependence of second harmonic resistance. Magnetic field dependence of $R^{2\omega}$ at each temperature measured under $I = 200$ μA. The blue, green and red regions correspond to normal, intermediate and superconducting regions, respectively.

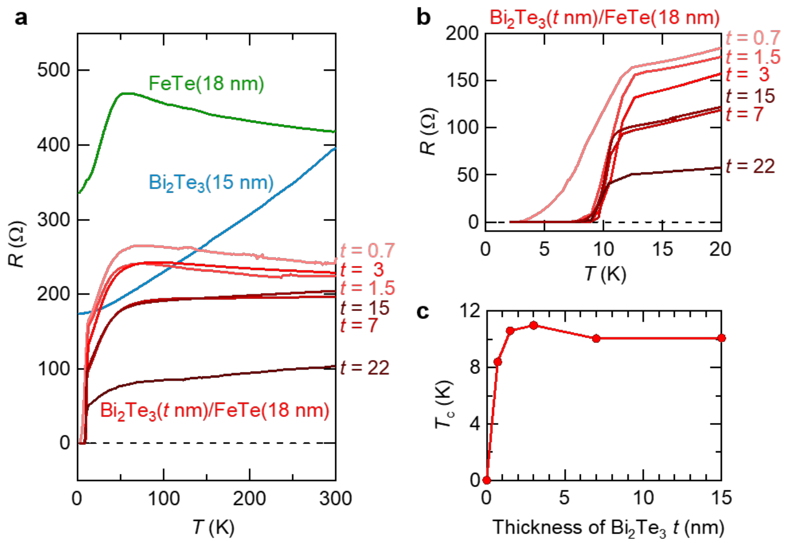

Supplementary Figure 4 | Thickness dependent transport property of Bi_2Te_3/FeTe. **a,** Temperature dependence of resistance of FeTe(18 nm) (green), Bi_2Te_3(15 nm) and Bi_2Te_3(*t* nm)/FeTe(18 nm) (red) thin films with different Bi_2Te_3 thickness. Each line corresponds to $t = 22$, 15, 7, 3, 1.5 and 0.7. Note that the thickness *t* represents an average thickness of Bi_2Te_3. **b,** Temperature dependence of resistance of Bi_2Te_3(*t* nm)/FeTe(18 nm) thin films with different Bi_2Te_3 thickness at low temperatures. **c,** Bi_2Te_3 thickness *t* dependence of superconducting transition temperature T_c in Bi_2Te_3(*t* nm)/FeTe(18 nm). T_c is defined as the temperature at the half of the normal resistance.

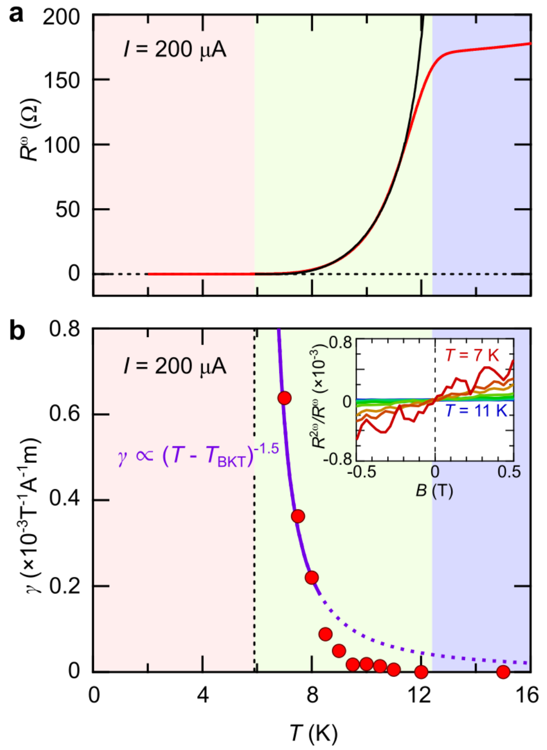

Supplementary Figure 5 | Temperature dependence of nonreciprocal transport in Bi_2Te_3(1.5 nm)/FeTe(18 nm). a, The temperature dependence of resistance measured under I = 200 μA. The black curve is the fitting of BKT transition using Halperin-Nelson formula, $R = R_0 \exp\left(-2b\left(\frac{T_{c0}-T}{T-T_{BKT}}\right)^{0.5}\right)$. The fitting gives the values, T_{c0} = 12.4 K and T_{BKT} = 5.9 K. The blue, green and red regions correspond to normal, intermediate and superconducting regions, respectively. **b,** The temperature dependence of γ value measured under I = 200 μA. The purple curve is the fitting with the formula $\gamma = \beta(T - T_{BKT})^{-1.5}$, where $\beta = 6.7 \times 10^{-4}$ $T^{-1}A^{-1}m$. Note that the BKT model and the fitting is valid only at around T_{BKT}, which is represented by the solid purple curve. The purple dotted curve is out of the applicable range of theory. The inset shows the magnetic field dependence of $R^{2\omega}/R^{\omega}$ measured under I = 200 μA at T = 7, 7.5, 8, 8.5, 9, 9.5, 10, 10.5 and 11 K.

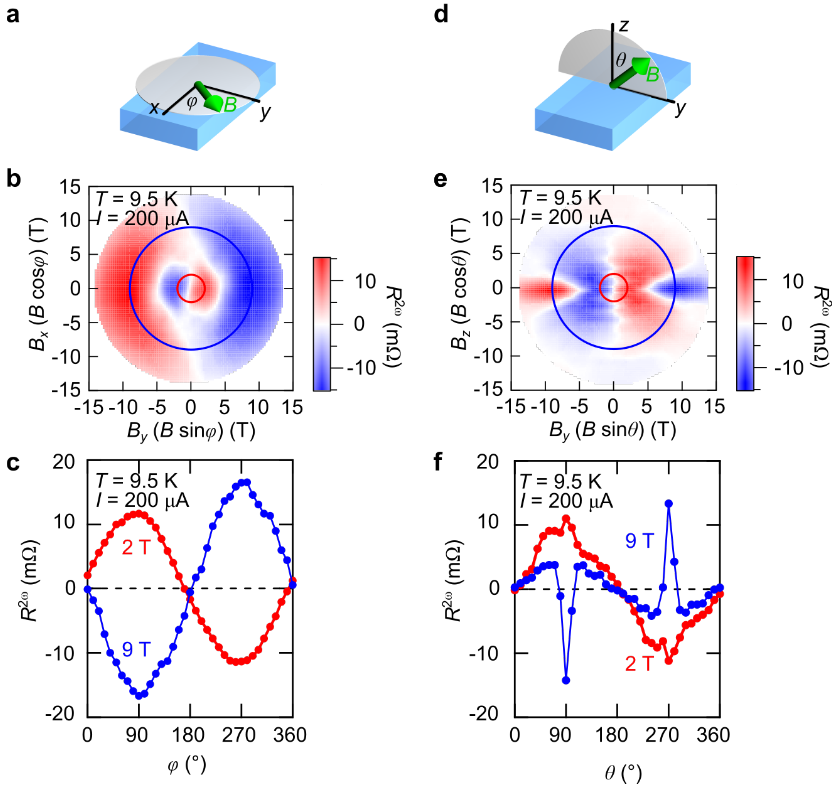

Supplementary Figure 6 | Angular dependence of second harmonic resistance. a, Schematic drawing of the magnetic field direction dependent measurement in *xy*-plane. **b,** The color plot of $R^{2\omega}$ under magnetic field in *xy*-plane. The horizontal (vertical) axis corresponds to *y* (*x*) component of the magnetic field B_y (B_x). The measurement is done at $T = 9.5$ K and $I = 200$ μA. **c,** The magnetic field direction dependence of $R^{2\omega}$ at $B =$ 2 T (red) and $B = 9$ T (blue) as indicated in the circles in **b**. **d-f,** The same as **a-c** for *yz*-plane.

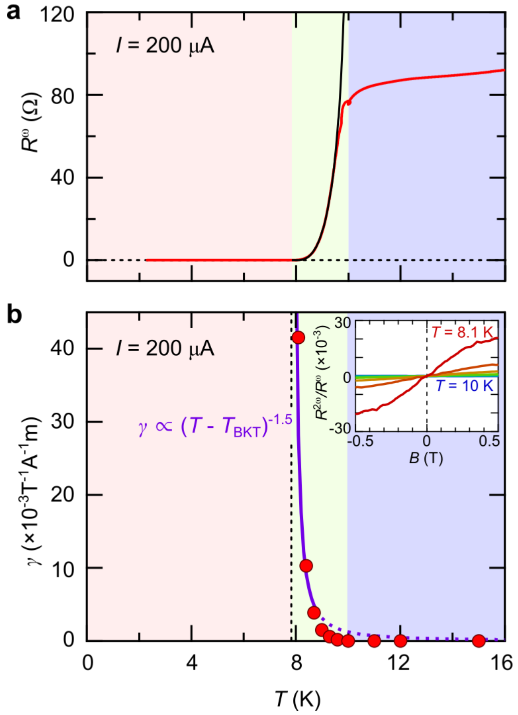

Supplementary Figure 7 | The reproducibility of the temperature dependence of nonreciprocal transport in Bi_2Te_3(15 nm)/FeTe(18 nm). a, The temperature dependence of resistance measured under I = 200 μA. The black curve is the fitting by BKT transition using Halperin-Nelson formula, $R = R_0 \exp\left(-2b\left(\frac{T_{c0}-T}{T-T_{\mathrm{BKT}}}\right)^{0.5}\right)$. The fitting gives the values, T_{c0} = 10.0 K and T_{BKT} = 7.8 K. The blue, green and red regions correspond to normal, intermediate and superconducting regions, respectively. **b,** The temperature dependence of γ value measured under I = 200 μA. The purple curve is the fitting with the formula $\gamma = \beta\,(T - T_{\mathrm{BKT}})^{-1.5}$, where β = 3.9 × 10^{-3} $T^{-1}A^{-1}m$, which is again much larger than that of Fig. S5. Note that the BKT model and the fitting is valid only at around T_{BKT}, which is represented by the solid purple curve. The purple dotted curve is out of the applicable range of theory. The inset shows the magnetic field dependence of $R^{2\omega}/R^{\omega}$ measured under I = 200 μA at T = 8.1, 8.4, 8.7, 9, 9.3, 9.6 and 10 K.

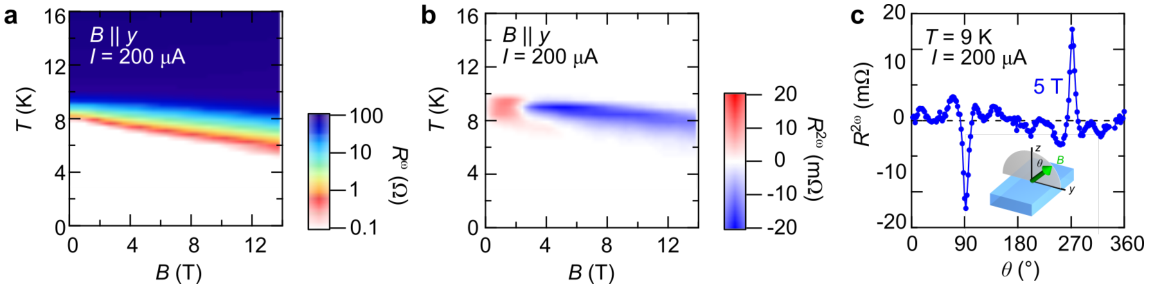

Supplementary Figure 8 | The reproducibility of the sign reversal of nonreciprocal transport at high fields in Bi_2Te_3(15 nm)/FeTe(18 nm). a, The contour plot of R^{ω} in the plane of in-plane magnetic field and temperature measured under $I = 200$ μA. **b,** The contour plot of $R^{2\omega}$ in the plane of in-plane magnetic field and temperature measured under $I = 200$ μA. **c,** The out-of-plane magnetic-field direction dependence of $R^{2\omega}$ at $B =$ 5 T within *zy* plane measured under $I = 200$ μA. θ is defined as an angle in *zy* plane measured from *z*-axis as shown in the inset.

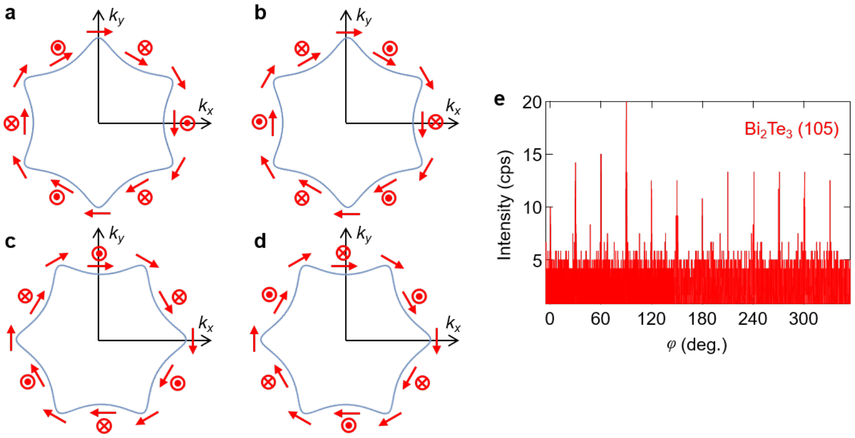

Supplementary Figure 9 | Twin domain formation and the Fermi surface of Bi_2Te_3 on FeTe. a-d, The Fermi surface and the spin texture of Bi_2Te_3 corresponding to each of the four types of twin domains. The in-plane and out-of-plane spin components are depicted by arrows and circular symbols, respectively. **e,** The azimuth angle φ dependence of X-ray diffraction on Bi_2Te_3 (105) reflection for Bi_2Te_3(15 nm)/FeTe(18 nm)/CdTe.

Supplementary Note 1 | Structural characterization of Bi_2Te_3/FeTe/CdTe

Supplementary Figure 1 shows the high-angle annular dark-field (HAADF) image taken by a scanning transmission electron microscopy (STEM) of Bi_2Te_3(15 nm)/FeTe(18 nm)/CdTe. Since Bi_2Te_3 has three-fold symmetry and FeTe has four-fold symmetry, the twin domain formation of Bi_2Te_3 is unavoidable as illustrated in Supplementary Figures 1b and c. Nevertheless, they make a sharp interface as shown in Supplementary Figure 1a probably due to the layered van der Waals nature of Bi_2Te_3 and FeTe. Besides, in the energy dispersive x-ray spectroscopy (EDX) images, the interdiffusion of the element at the interface between Bi_2Te_3 and FeTe is not discerned. Thus, the effect of the magnetic impurities to the topological surface states is negligible.

Supplementary Note 2 | Characterization of superconductivity

We characterize the nature of superconductivity from the resistance measurement. Supplementary Figure 2a shows the temperature dependence of resistance in Bi_2Te_3/FeTe. The gradual decrease of resistance is observed at around the superconducting onset temperature. This feature is well reproduced with the Aslamazov-Larkin pair contribution to conductivity. The appearance of fluctuating Cooper pairs leads to the initiation of a new conducting channel for charge transport, leading to a decrease of resistance as follows[1]:

$$R(T) = \left(\frac{1}{R_{\mathrm{N}}(T)} + \Delta G_{\mathrm{AL}}\right)^{-1}, \tag{1}$$

where $R_{\mathrm{N}}(T)$ is the temperature dependence of normal resistance and ΔG_{AL} is the excess conductance due to the emerging superconducting channel. We fit the resistance curve by

$$R_{\mathrm{N}}(T) = a + cT, \tag{2}$$

$$\Delta G_{\mathrm{AL}} = \frac{e^2}{16\hbar}\left(\frac{T_{\mathrm{c0}}}{T_{\mathrm{c0}} - T}\right),$$

where T_{c0} the temperature at which the finite amplitude of the order parameter develops. The blue dotted curve in Supplementary Figure 2a is the fitting by the Aslamazov–Larkin formula, which gives a = 78.1 Ω, c = 1.93 Ω/T and $T_{\mathrm{c0}} = 10.7$ K. On the other hand, the finite resistance at low temperature region is explained in terms of the vortex flow above the Berezinskii-Kosterlitz-Thouless (BKT) transition temperature at which zero-resistance value is realized by the binding of the vortex-antivortex pair. We fit the low temperature region in terms of the BKT transition using the Halperin-Nelson formula[2-4].

$$R(T) = R_0 \exp\left(-2b\left(\frac{T_{c0}-T}{T-T_{\mathrm{BKT}}}\right)^{0.5}\right), \tag{3}$$

where R_0 and b are material parameters and T_{BKT} is the BKT transition temperature. The fitting gives $R_0 = 236\ \Omega$, $b = 1.5$ and $T_{\mathrm{BKT}} = 8.0$ K. The well fitted curve by the Halperin-Nelson formula confirms the two-dimensional nature of superconductivity as discussed in the literature[5]. Note that the BKT transition temperature is different from that derived from Fig. 2d in the main text, where $T_{\mathrm{BKT}} = 6.0$ K. The difference in temperature comes from the difference in the applied current. In Fig. 2d, I = 200 μA is applied to obtain the high S/N ratio for the nonreciprocal measurement, the large applied current caused heating and lowered the BKT transition temperature. The divergent behavior of γ is, however, unchanged by the decrease of T_{BKT}.

Supplementary Figure 1b shows the current-voltage (I-V) characteristics. The I-V characteristics are linear in the normal region (T = 12 K). The power α of I-V characteristics $V = |I|^{\alpha}$ changes as a function of the temperature. In the log-log scale, the power α can be extracted from the slope of the curve. The extracted temperature dependence of the power is plotted in Supplementary Figure 2c. The jump in the power α from 1 to 3 is observed, which is characteristic of the two-dimensional superconductivity[4]. α = 3 corresponds to the BKT transition temperature $T_{\mathrm{BKT}} = 7.9$ K, which well coincides with the BKT transition temperature estimated from the temperature dependence of resistance value.

Supplementary Note 3 | Derivation of the second harmonic resistance

Here, we derive the expression for the second harmonic resistance from equation (1) in the main text. As mentioned in the main text, when the resistance value is dependent on the current direction, $R = R_0(1+\gamma BI)$, the nonreciprocal current comes from the second term and this can be measured with second harmonic voltage measurement. When ac excitation current of $I = \sqrt{2}I_0 \sin\omega t$ is applied to the sample. The voltage can be expressed as follows:

$$\begin{aligned} V &= R_0 I(1+\gamma BI) \\ &= \sqrt{2}R_0 I_0 \sin\omega t - \gamma B R_0 I_0^2 \cos 2\omega t + \gamma B R_0 I_0^2 \end{aligned} \tag{S4}$$

Consequently, the amplitude of the first harmonic and second harmonic resistance becomes $R^{\omega} = R_0$ and $R^{2\omega} = \gamma B R_0 I_0/\sqrt{2}$, respectively.

Supplementary Note 4 | Temperature dependence of second harmonic resistance

Supplementary Figure 3 displays the temperature dependence of $R^{2\omega}$. In the normal region (blue), $R^{2\omega}$ is not observed within the measurement noise level. In the superconducting region (red), $R^{2\omega}$ vanishes with the disappearance of R^{ω}. γ cannot be defined in this region because $R^{2\omega}/R^{\omega}$ becomes 0/0. $R^{2\omega}$ is finite only in the intermediate region (green). Although $R^{2\omega}$ decreases toward zero as the temperature approaches T_{BKT}, γ diverges toward T_{BKT} because R^{ω} goes more rapidly to zero.

Supplementary Note 5 | Thickness dependent transport property of Bi_2Te_3/FeTe thin films

Supplementary Figure 4 shows the thickness dependence of the transport property of Bi_2Te_3(t nm)/FeTe(18 nm) thin films. As shown in Supplementary Figure 4a, the resistance of the normal state basically decreases as Bi_2Te_3 thickness t becomes thicker because of the increased bulk conductivity. The superconducting transition is observed in all the samples with various thickness (Supplementary Figure 4b). The superconducting transition temperature determined from the half resistance of the normal state is summarized in Supplementary Figure 4c. Since the thickness t represents an average thickness of Bi_2Te_3, t = 0.7 means that the film consists of the FeTe area covered with monolayer Bi_2Te_3 (1QL, or 1 nm) and the area without Bi_2Te_3. The fact that T_c of t = 0.7 sample takes the intermediate value of t = 0 and t = 1.5 in the broad transition behavior, probably means that the area covered with Bi_2Te_3 shows superconductivity while the area without Bi_2Te_3 does not show superconductivity

Supplementary Note 6 | Temperature dependence of second harmonic resistance in Bi_2Te_3(1.5 nm)/FeTe(18 nm)

Here, we show the temperature dependence of the second harmonic resistance of Bi_2Te_3(1.5 nm)/FeTe(18 nm) and discuss its implication in detail. When the Bi_2Te_3 film is as thin as 1 QL or 2 QL, the quantum tunneling between the top and bottom surfaces are strong enough to open a hybridization gap at the Dirac cone, while the surface state is still conductive because of the n-type doped nature of Bi_2Te_3 (Ref. 6). It is theoretically and experimentally shown that the spin polarization of the surface state is suppressed in the ultrathin limit because of the mixing with the opposite spin component from the other surface[7]. Thus, if the nonreciprocal signal decreases in the ultrathin limit, it strongly supports

the surface state origin of nonreciprocal transport. From Supplementary Figure 5a, the BKT transition temperature of the sample is estimated to be T_{BKT} = 5.9 K, which is almost the same as Fig. 2d in the main text. On the other hand, we observe a sizable difference in γ values. The γ value in Bi_2Te_3(1.5 nm)/FeTe(18 nm) in Supplementary Figure 5b is about an order of magnitude smaller than Bi_2Te_3(15 nm)/FeTe(18 nm) in Fig. 2e in the main text. The fitting by the formula $\gamma = \beta(T - T_{BKT})^{-1.5}$ gives $\beta = 6.7 \times 10^{-4}$ $T^{-1}A^{-1}m$ in Bi_2Te_3(1.5 nm)/FeTe(18 nm), which is an order of magnitude smaller than $\beta = 5.3 \times 10^{-3}$ $T^{-1}A^{-1}m$ in Bi_2Te_3(15 nm)/FeTe(18 nm). Although finite second harmonic resistance is still observed due to the remaining spin polarization[7], an order of magnitude suppression of the signal in Bi_2Te_3(1.5 nm)/FeTe(18 nm) demonstrates the surface state origin of nonreciprocal transport.

Supplementary Note 7 | Further analysis of angular dependence of second harmonic resistance

Supplementary Figure 6 displays the angular dependence of $R^{2\omega}$, which helps to understand the discussion in the main text. In *xy*-plane (Supplementary Figures 6a-c), the signal shows sinφ dependence for all the magnetic field region. On the other hand, in *yz*-plane (Supplementary Figures 6d-f), the signal does not follow sinθ dependence. The sign reversal appears only in the small pocket, where the high magnetic field is aligned almost perfectly to the in-plane direction.

Supplementary Note 8 | Reproducibility of the temperature and angular dependence of second harmonic resistance in another sample

Here, we show the reproducibility of the temperature and angular dependence of $R^{2\omega}$ in another Bi_2Te_3(15 nm)/FeTe(18 nm) sample. As shown in Supplementary Figure 7a, the temperature dependence of resistance is well-fitted by Halperin-Nelson formula, which gives the BKT transition temperature of T_{BKT} = 7.8 K. In Supplementary Figure 7b, γ value is finite in intermediate region and diverges as the temperature approaches to T_{BKT} in a similar way to Fig. 2e in the main text. The reproducibility of the negative component of $R^{2\omega}$ at high fields and its high sensitivity to magnetic field direction is shown in Supplementary Figure 8. In a similar way to Fig. 3c in the main text, we observe a positive component at low fields and a negative component at high fields. The negative component in Supplementary Figure 8b is extended to the lower fields compared with Fig. 3c. This is probably because the relative magnitude of the positive and negative components depends on the sample reflecting the

different origins of the two components. In addition, the negative component appears only at around 90° and 270° as shown in Supplementary Figure 8c in a similar manner to Fig. 3f in the main text.

Supplementary Discussion 1 | Discussion on vanishingly small $R^{2\omega}$ under perpendicular magnetic field

The $R^{2\omega}$ signal is not observed under the perpendicular magnetic field as shown in Fig. 3f in the main text. We attribute this to the formation of twin domains of Bi_2Te_3. Bi_2Te_3 has a rhombohedral crystal structure with a space group of $R\bar{3}m$. The three-fold symmetry along the *c*-axis causes the hexagonal warping at the surface state[8]. Here, in addition to the in-plane spin momentum locking, the surface state possesses the perpendicular spin component for some specific momentum directions as shown in Supplementary Figure 9a. Thus, if we apply the current along the specific crystallographic axes, the $R^{2\omega}$ signal appears under the perpendicular magnetic field as well as under the in-plane magnetic field[9]. Since the $R^{2\omega}$ signal under the perpendicular magnetic field comes from the hexagonal warping, it appears only when the twin domain formation is suppressed, which can be achieved by using the lattice-matched substrate with three-fold symmetry substrate such as InP (111) (Ref. 10). In the present experiment, however, Bi_2Te_3 with three-fold symmetry is grown on FeTe with four-fold symmetry, resulting in the formation of four types of twin domains of Bi_2Te_3. The TEM image clearly shows such twin domain formation (Supplementary Figures 1a-c). This is also evidenced in the observation of 12 peaks in the azimuth angle scan of Bi_2Te_3 (105) reflection (Supplementary Figure 9e). The corresponding Fermi surface of each domain is shown in Supplementary Figures S9a-d. Here, when the current is applied along k_x-direction, the contribution from the perpendicular spin component is canceled out in Supplementary Figures S9a and S9b. Incidentally, the perpendicular spin component does not exist in Supplementary Figures S9c and S9d. Thus, $R^{2\omega}$ signal under the perpendicular magnetic field is expected to become zero in Bi_2Te_3/FeTe, which is consistent with the experimental result.

Supplementary References

1. Aslamazov, L. G. & Larkin, A. I. The influence of fluctuation pairing of electrons on the conductivity of normal metal. *Phys. Lett.* **26A**, 238–239 (1968).
2. Berezinskii, V. L. Destruction of long-range order in one-dimensional and two-dimensional systems possessing a continuous symmetry group. II. Quantum systems. *Sov. Phys. JETP* **34**, 610–616 (1972).
3. Kosterlitz, J. M. & Thouless, D. J. Ordering, metastability and phase transitions in two-dimensional systems. *J. Phys. C* **6**, 1181–1203 (1973).
4. Halperin, B. I. & Nelson, D. R. Resistive transition in superconducting films. *J. Low Temp. Phys.* **36**, 599–616 (1979).
5. He, Q. L. *et al.* Two-dimensional superconductivity at the interface of a Bi_2Te_3/FeTe heterostructure. *Nature Commun.* **5**, 4247 (2014).
6. Li, Y.-Y. *et al.* Intrinsic Topological Insulator Bi_2Te_3 Thin Films on Si and Their Thickness Limit. *Adv. Mater.* **22**, 4002–4007 (2010).
7. Neupane, M. *et al*. Observation of quantum-tunnelling-modulated spin texture in ultrathin topological insulator Bi_2Se_3 films. *Nat. Commun.* **5**, 3841 (2014).
8. He, P. *et al*. Bilinear magnetoelectric resistance as a probe of three-dimensional spin texture in topological surface states. *Nature Phys.* **14**, 495–499 (2018).
9. Fu, L. Hexagonal Warping Effects in the Surface States of the Topological Insulator Bi_2Te_3. *Phys. Rev. Lett.* **103**, 266801 (2009).
10. Richardella, A. *et al*., Characterizing the structure of topological insulator thin films. *APL Mater.* **3**, 083303 (2015).
