# Supplementary material for: Nonreciprocal charge transport at topological insulator/superconductor interface
Source: Nat Commun. 2019 Jun 21;10:2734. doi: 10.1038/s41467-019-10658-3 (PMC6588591; doi:10.1038/s41467-019-10658-3)
Supplement: Supplementary file 2 — Peer Review File [file 41467_2019_10658_MOESM2_ESM.pdf]

Editorial Note: This manuscript has been previously reviewed at another journal that is not operating a transparent peer review scheme. The manuscript was considered suitable for publication without further review at Nature Communications .
